# Coicis semen protects against focal cerebral ischemia-reperfusion injury by inhibiting oxidative stress and promoting angiogenesis via the TGFβ/ALK1/Smad1/5 signaling pathway

**DOI:** 10.18632/aging.202194

**Published:** 2020-11-16

**Authors:** Jin Du, Guobing Yin, Yida Hu, Si Shi, Jiazhen Jiang, Xiaoyan Song, Zhetao Zhang, Zeyuan Wei, Chaoliang Tang, Haiyan Lyu

**Affiliations:** 1 Department of Neurosurgery, The People’s Hospital of Chizhou, Chizhou 247000, Anhui, China; 2 Department of Anesthesiology, The First Affiliated Hospital of USTC, Division of Life Sciences and Medicine, University of Science and Technology of China, Hefei 230001, Anhui, China; 3 Department of Anesthesiology, Renmin Hospital of Wuhan University, Wuhan 430060, Hubei, China; 4 Department of Emergency, Huashan Hospital North, Fudan University, Shanghai 201907, China; 5 Department of Neurology, Shanghai General Hospital, Shanghai Jiao Tong University School of Medicine, Shanghai 201620, China; 6 Department of Pharmacy, The First Affiliated Hospital of USTC, Division of Life Sciences and Medicine, University of Science and Technology of China, Hefei 230036, Anhui, China

**Keywords:** ischemic stroke, ROS, angiogenesis, TGFβ/ALK1 signaling pathway, therapy

## Abstract

Background: Ischemic stroke is a devastating disease that causes long-term disability. However, its pathogenesis is unclear, and treatments for ischemic stroke are limited. Recent studies indicate that oxidative stress is involved in the pathological progression of ischemic stroke and that angiogenesis participates in recovery from ischemic stroke. Furthermore, previous studies have shown that Coicis Semen has antioxidative and anti-inflammatory effects in a variety of diseases. In the present study, we investigated whether Coicis Semen has a protective effect against ischemic stroke and the mechanism of this protective effect.

Results: Coicis Semen administration significantly decreased the infarct volume and mortality and alleviated neurological deficits at 3, 7 and 14 days after MCAO. In addition, cerebral edema at 3 days poststroke was ameliorated by Coicis Semen treatment. DHE staining showed that ROS levels in the vehicle group were increased at 3 days after reperfusion and then gradually declined, but Coicis Semen treatment reduced ROS levels. The levels of GSH and SOD in the brain were increased by Coicis Semen treatment, while MDA levels were reduced. Furthermore, Coicis Semen treatment decreased the extravasation of EB dye in MCAO mouse brains and elevated expression of the tight junction proteins ZO-1 and Occludin. Double immunofluorescence staining and western blot analysis showed that the expression of angiogenesis markers and TGFβ pathway-related proteins was increased by Coicis Semen administration. Consistent with the *in vivo results*, cytotoxicity assays showed that Coicis Semen substantially promoted HUVEC survival following OGD/RX *in vitro*. Additionally, though LY2109761 inhibited the activation of TGFβ signaling in OGD/RX model animals, Coicis Semen cotreatment markedly reversed the downregulation of TGFβ pathway-related proteins and increased VEGF levels.

Methods: Adult male wild-type C57BL/6J mice were used to develop a middle cerebral artery occlusion (MCAO) stroke model. Infarct size, neurological deficits and behavior were evaluated on days 3, 7 and 14 after staining. In addition, changes in superoxide dismutase (SOD), GSH and malondialdehyde (MDA) levels were detected with a commercial kit. Blood-brain barrier (BBB) permeability was assessed with Evans blue (EB) dye. Western blotting was also performed to measure the levels of tight junction proteins of the BBB. Additionally, ELISA was performed to measure the level of VEGF in the brain. The colocalization of CD31, angiogenesis markers, and Smad1/5 was assessed by double immunofluorescent staining. TGFβ pathway-related proteins were measured by western blotting. Furthermore, the cell viability of human umbilical vein endothelial cells (HUVECs) following oxygen-glucose deprivation/reoxygenation (OGD/RX) was measured by Cell Counting Kit (CCK)-8 assay.

Conclusions: Coicis Semen treatment alleviates brain damage induced by ischemic stroke through inhibiting oxidative stress and promoting angiogenesis by activating the TGFβ/ALK1 signaling pathway.

## INTRODUCTION

Ischemic stroke is the single most devastating neurological disease and a leading cause of death worldwide [1]. Clinical treatment by means of tissue plasminogen activator (tPA)-mediated thrombolysis and endovascular thrombectomy has significantly improved options within the acute time window of ischemic attack. In rodent middle cerebral artery occlusion (MCAO) models, the injury is characterized by the necrosis of neurons of the cerebral cortex caused by the combination of extensive inflammation and oxidative stress [2]. Moreover, cumulative evidence has shown that the consumption of antioxidants and overproduction of reactive oxygen species (ROS) are associated with the severity and progression of CIRI [3]. Although the neuroprotective effects of a great deal of antioxidant agents have been identified in rodent models, most have failed in subsequent clinical trials. Thus, explorations of the in-depth neuropathological mechanisms of ischemic stroke and the development of new therapeutic strategies for ischemic stroke present great challenges.

Coicis Semen, a polyphenolic compound extracted from Coix, is widely consumed as food and medicine in Asian countries. As a traditional Chinese medicine, Coicis Semen is thought to possess antioxidant, immunological, and anti-inflammatory effects and has been used to treat diabetic nephropathy [4], gastrointestinal diseases [5], skin diseases [6], and even tumors [7–9]. However, whether Coicis Semen exerts a neuroprotective effect against ischemic stroke needs to be investigated.

Angiogenesis is the physiological process by which new microvessels branching from pre-existing vessels are formed [10]. After ischemic stroke, the blood-brain barrier (BBB) is acutely disrupted, resulting in secondary brain injury due to an increase in the degree of cerebral edema, causing the infiltration of peripheral immune cells and exacerbating the inflammatory response in the central nervous system (CNS) [11]. Accumulating evidence indicates that angiogenesis not only alleviates BBB leakage [12] but also contributes to neuronal metabolism and remodeling [13]. In rodent MCAO models, endothelial cells (ECs) start to proliferate as early as 12-24 h after ischemia, but the reformation of cerebral microvessels in the penumbra and core of the ischemic infarct occurs within 4 to 7 days [14, 15]. Meanwhile, neuronal remodeling and axonal outgrowth do not occur until 14 days after stroke [15]. Thus, the promotion of angiogenesis may become a promising therapeutic strategy for the restorative treatment of ischemic stroke. Transforming growth factor-β (TGF-β) signaling pathways are involved in regulating EC proliferation and angiogenesis [16, 17]. Activin receptor-like kinase-1 (ALK1) is a surface receptor for the TGF-β1 protein that is preferentially expressed in ECs. During angiogenesis, ALK-1 is activated by binding TGF-β, which then induces the phosphorylation of Smad1/5/8, which dimerize with Smad4; this complex is then transferred to the nucleus, where it regulates the transcription of angiogenesis-related genes [18, 19]. Several studies found that the deletion of any components of the TGF-β1/ALK1/Smad1/5/8 pathway resulted in abnormalities in primitive vascular plexus formation and embryonic lethality in mice due to defects in angiogenesis [20, 21]. In addition, studies have demonstrated that the TGF-β/ALK5 signaling pathway plays a critical role in neurogenesis and functional recovery following ischemic stroke [22, 23]. Thus, these observations led us to wonder whether Coicis Semen can regulate angiogenesis via the TGF-β1/ALK1/Smad1/5/8 signaling pathway following stroke, which remains unknown.

In this study, we generated a mouse MCAO model and oxygen-glucose deprivation/reoxygenation (OGD/RX) model in human umbilical vein endothelial cells (HUVECs) to investigate whether Coicis Semen has a neuroprotective effect and its potential molecular effects on ischemia-induced oxidative stress and angiogenesis. Our results demonstrate that Coicis Semen may protect against cerebral ischemic injury in the brain by inhibiting oxidative stress and promoting angiogenesis via activation of the TGFβ1/ALK-1/Smad1/5/8 signaling pathway.

## RESULTS

### Coicis semen reduced infarct volume, ameliorated neurological deficits and reduced edema after 3 days of MCAO

**Figure 1:**
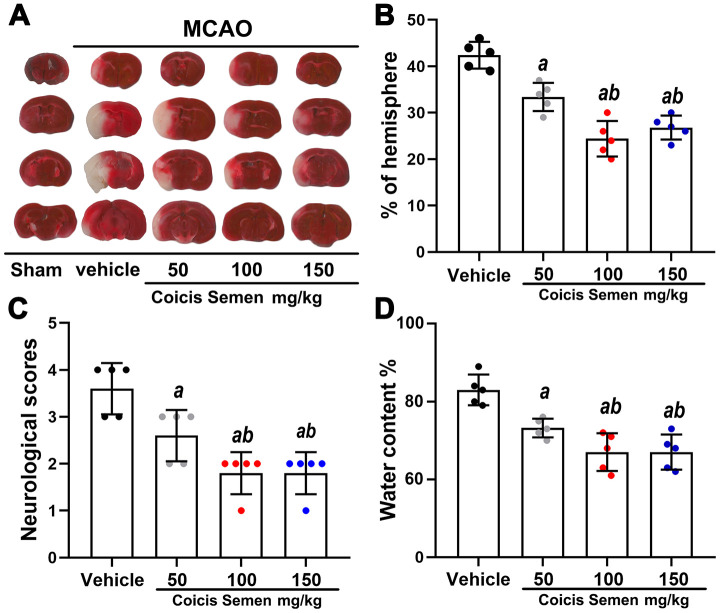
**Coicis semen treatment reduced the infarct volume, improved neurological function and decreased the brain water content in MCAO mice.** (**A**) TTC staining of brain sections from vehicle- or Coicis Semen-treated mice taken at 3 days poststroke. (**B**, **C**, **D**) Effects of Coicis Semen at different concentration on infarct volume, neurological score and brain water content in mice at 3 days poststroke. Mean ± SD. n = 5. *^a^P* < 0.05 vs. vehicle; *^b^P* < 0.05 vs. Coicis Semen (50 mg/kg). Statistical analyses were performed by one-way ANOVA followed by Tukey’s multiple comparison tests.

To examine whether Coicis Semen has a neuroprotective effect and determine its optimal dose in MCAO mice, we established a mouse MCAO model and measured infarct volume, neurological deficit outcomes and the water content of the brain. Neurological scoring was performed 3 days after MCAO. TTC staining of all brain slices showed that the administration of Coicis Semen (50, 100, and 150 mg/kg) significantly reduced infarct volume compared with that of the MCAO + vehicle group (*P* < 0.05) (Figure 1A, 1B). Similarly, neurological scores and the cerebral water content were significantly reduced in the Coicis Semen-treated groups compared with the vehicle-treated group (*P* < 0.05) (Figure 1C, 1D). Additionally, Coicis Semen at a dose of 100 mg/kg dose had the greatest neuroprotective effect, and this dose was selected for subsequent experiments.

### Coicis semen promoted long-term neurological recovery after ischemic stroke

**Figure 2:**
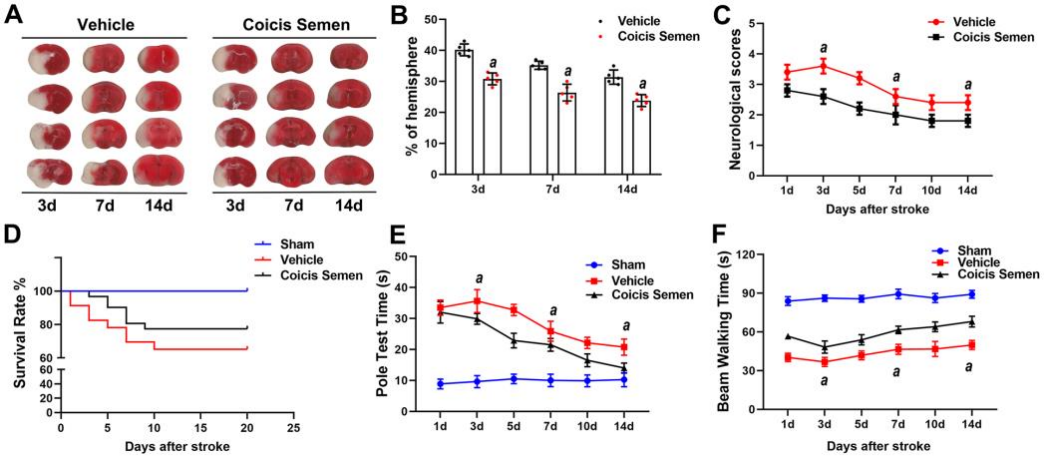
**Coicis semen treatment promoted long-term neurological recovery after ischemic stroke.** (**A**) The ipsilateral infarct volume was detected at 3, 7 and 14 days after stroke by using TTC staining and then normalized to the infarct volume of the contralateral hemisphere to correct for edema; the data are expressed as a percentage. (**B**) Bar graphs show quantified infarct volumes. n =5, *^a^P* < 0.05 vs. vehicle. Statistical analyses were performed by two-tailed Student’s *t*-test. (**C**) Neurological deficit scores. n =5. (**D**) Survival rate. n = 15-20 (**E**) Pole test time. (**F**) Beam walking time. Mean ± SD. n = 10-12. *^a^P* < 0.05 vs. vehicle by one-way ANOVA followed by Tukey’s multiple comparison tests (individual time points).

Although ischemic injury induces neurological function deficits in the acute stage of stroke, recovery after the injury is possible. Therefore, we explored the long-term neuroprotective effect of Coicis Semen on stroke. We detected the relative infarct volume, survival rate, neurological scores and behavioral functions of the MCAO mice on days 3, 7 and 14 after MCAO. Compared with vehicle administration, Coicis Semen administration significantly reduced the relative infarct volume at each time point (*P*< 0.05, Figure 2A, 2B). Moreover, the neurological scores were also significantly decreased on days 3, 7, and 14 after MCAO mice were treated with Coicis Semen (*P*< 0.05, Figure 2C). With prolonged reperfusion, the neurological function of mice in the MCAO + vehicle group gradually recovered. Additionally, the survival rates of the Coicis Semen groups were remarkably elevated compared with that in the MCAO + vehicle group (Figure 2D). Furthermore, Coicis Semen significantly reduced motor deficit, as shown by the increase in the latency to fall from the rotarod and pole at each time point after stroke compared with that in the MCAO + vehicle group (*P*< 0.05, Figure 2E, 2F).

### Coicis semen decreased oxidative stress following ischemic stroke

**Figure 3:**
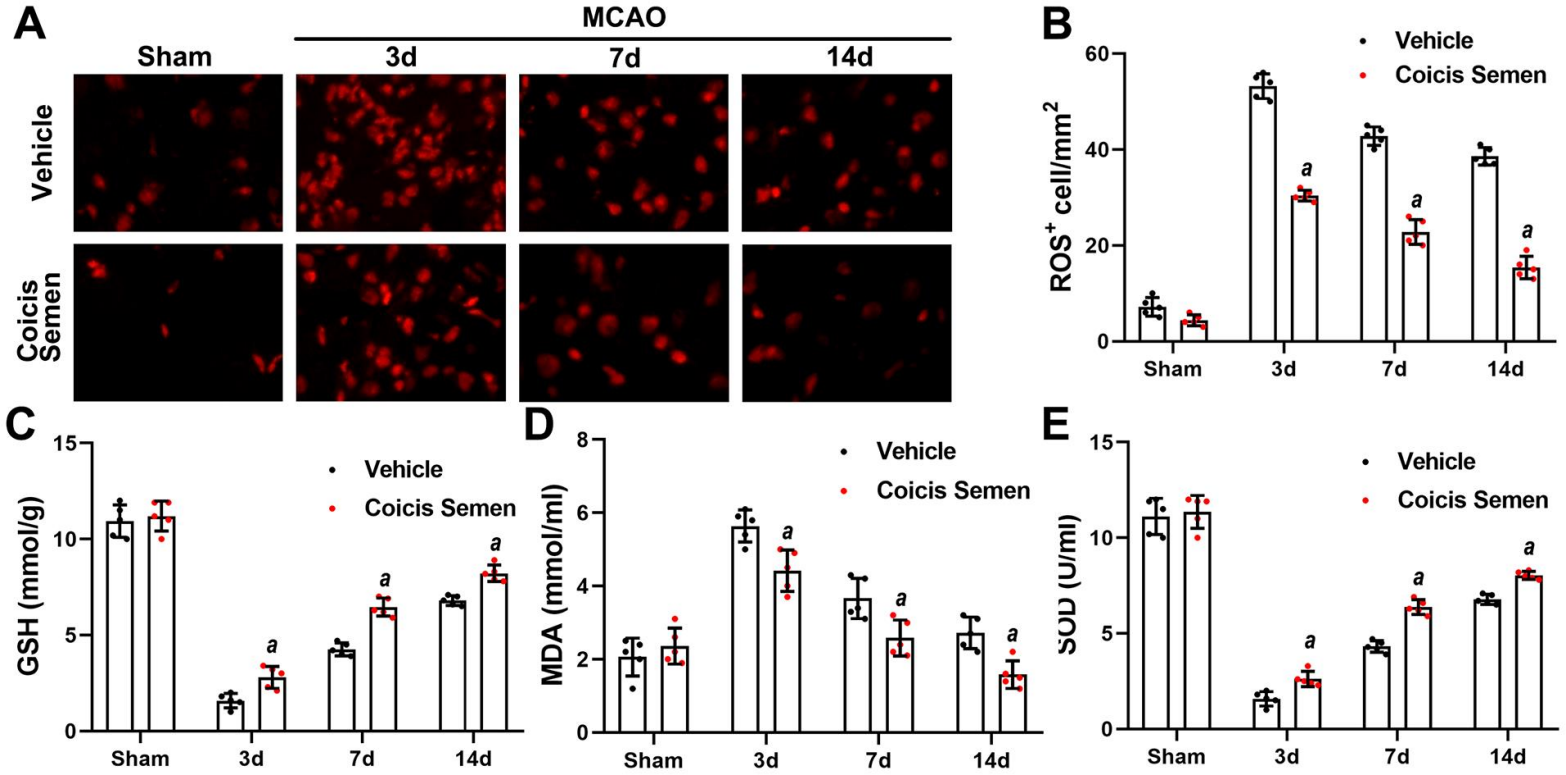
**Coicis semen treatment reduced oxidative stress levels after ischemic stroke.** (**A**) Changes in the production of ROS were revealed by DHE staining. Scale bar =50 μm. (**B**) Quantification of ROS-positive cells in the brain. n = 5, *^a^P* < 0.05 vs. vehicle. Statistical analyses were performed by two-tailed Student’s *t*-test. (**C**, **D**, **E**) The levels of GSH, MDA and SOD were quantified by using commercial kits. Mean ± SD. n = 5. *^a^P* < 0.05 vs. vehicle. Statistical analyses were performed by two-tailed Student’s *t*-test.

To understand the neuroprotective mechanism of Coicis Semen, we focused on its antioxidation effect. After stroke, oxidative stress is activated, and a large amount of ROS is produced. SOD, GSH, and MDA are vital biomarkers of oxidative stress, and their levels indirectly reflect the degree of lipid peroxidation and directly show the level of free radical scavengers. We found that the production of ROS in the brain was markedly increased at 3 days after stroke and had gradually decreased on the 7^th^ and 14^th^ days following stroke. Furthermore, Coicis Semen treatment significantly decreased the number of ROS-positive cells compared to those in the MCAO + vehicle group (*P*< 0.05, Figure 3A, 3B). The Coicis Semen-treated groups showed reduced levels of MDA in comparison to the MCAO + vehicle group (*P*< 0.05, Figure 3C). Meanwhile, SOD and GSH levels were remarkably lower in the MCAO + vehicle group than in the Coicis Semen-treated groups (*P*< 0.05, Figure 3D, 3E). Taken together, these data suggest that Coicis Semen can inhibit oxidative stress activation in ischemic stroke.

### Coicis semen attenuated ischemic stroke-induced BBB damage

**Figure 4:**
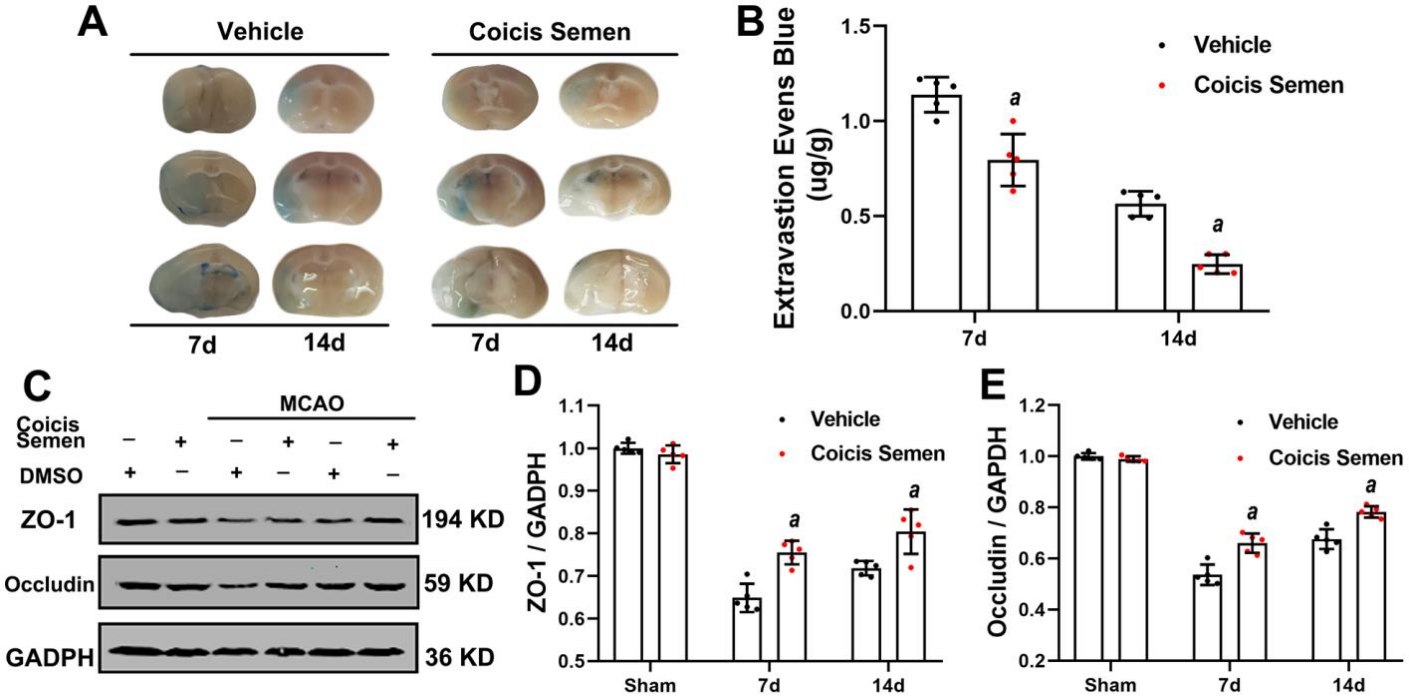
**Coicis semen alleviated blood-brain barrier (BBB) leakage after ischemic stroke.** (**A**) Images showing Evans blue (EB) dye leakage in brains after EB injection (by tail vein). (**B**) EB dye extravasation 2 h after tail vein injection of EB dye was quantitatively analyzed using a spectrophotometer. (**C**) Western blot analysis showing that Coicis Semen treatment increased the expression of ZO-1 and Occludin. (**D**) Quantitative analysis of the ZO-1 protein levels. (**E**) Quantitative analysis of the Occludin protein levels. Mean ± SD. n = 5. *^a^P* < 0.05 vs. vehicle. Statistical analyses were performed by two-tailed Student’s *t*-test.

Since the BBB plays a crucial role in the long-term effects of ischemic stroke, we investigated the effect of Coicis Semen on the recovery of BBB function. On days 7 and 14 after MCAO, we evaluated the BBB permeability of mice by EB dye staining. Under normal conditions, EB dye cannot enter the brain due to the protection conferred by the BBB. More whole-brain vascular leakage was found in mice in the MCO + vehicle group than in mice in the Coicis Semen-treated groups (Figure 4A). Moreover, EB extravasation was reduced in the Coicis Semen-treated groups at each time point after stroke compared with that in the MCAO + vehicle group (*P*< 0.05, Figure 4B). The BBB is composed of ECs, astrocytic end-feet, pericytes, and tight junction proteins, including ZO-1 and Occludin. Thus, we used western blot analysis to detect the expression of ZO-1 and Occludin, the results of which showed that the levels of both ZO-1 and occludin were significantly increased in Coicis Semen-treated MCAO mice compared with vehicle-treated MCAO mice (*P*< 0.05, Figure 4C–4E).

### Coicis semen promoted angiogenesis in the ischemic penumbra of MCAO mice

**Figure 5:**
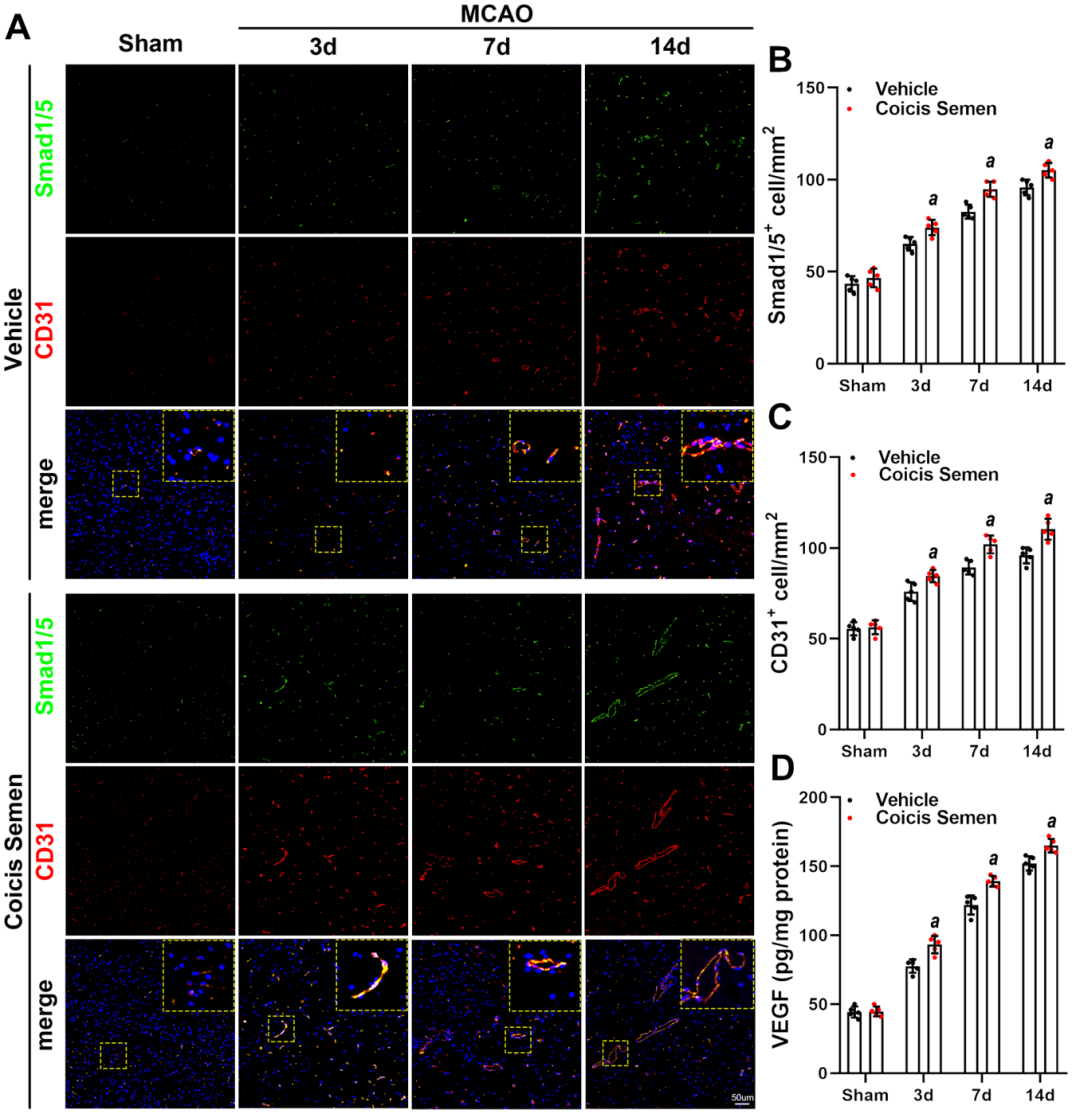
**Coicis semen promoted angiogenesis after ischemic stroke.** (**A**) Images showing CD31 detection in the ischemic penumbra via immunofluorescent staining and counterstaining with DAPI. (**B**) Quantification of CD31-positive cells in the ischemic penumbra. (**C**) VEGF levels were detected by ELISA. Mean ± SD. n = 5. *^a^P* < 0.05 vs. vehicle. Statistical analyses were performed by two-tailed Student’s *t*-test.

The improvement in stroke outcomes in Coicis Semen-treated mice led to us to hypothesize that Coicis Semen improves angiogenesis and cerebral blood flow during stroke recovery. To test this hypothesis, we measured expression of the angiogenesis-related proteins CD31 and VEGF using immunofluorescence and ELISA. The immunofluorescence results showed that Coicis Semen treatment significantly increased the proportion of CD31-positive cells in the penumbra of the ischemic brain compared to that in the MCAO + vehicle group after 3, 7, or 14 days of reperfusion after MCAO (*P*< 0.05, Figure 5A, 5B). Furthermore, Coicis Semen administration also considerably improved VEGF levels in the penumbra of the ischemic brains of MCAO mice at 3, 7 and 14 days after reperfusion following MCAO compared to those in the ischemic brains of vehicle-treated mice (*P*< 0.05, Figure 5C).

### Coicis semen potentiated TGF-β1/ALK1/Smad1/5 signaling pathway activation after stroke

**Figure 6:**
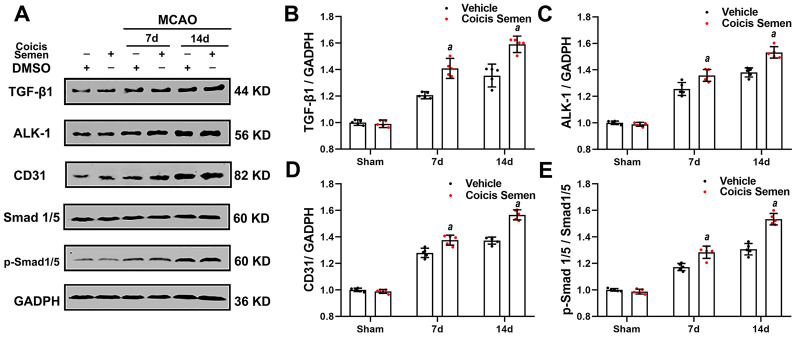
**Coicis semen treatment activated the TGF-β1-ALK1-Smad1/5 signaling pathway after stroke.** (**A**) Western blot showing that Coicis Semen treatment increased the expression and phosphorylation of the TGF-β1-ALK1-Smad1/5-associated proteins TGF-β1, ALK-1, Smad 1/5, p-Smad 1/5 and CD31 at 7 and 14 days after stroke. (**B**–**E**) Quantitative analysis of the protein levels of TGF-β1, ALK-1, p-Smad 1/5 and CD31 in ischemic cortical tissue. Mean ± SD. n = 5. *^a^P* < 0.05 vs. vehicle. Statistical analyses were performed by two-tailed Student’s *t*-test.

To further explore the molecular signaling mechanism by which Coicis Semen attenuated ischemic-induced BBB injury and promoted angiogenesis, we measured the expression of members of the TGF-β1/ALK1/Smad1/5 signaling pathway, a key intracellular signaling system that plays a pivotal role in the proliferation of the endothelium and regulation of angiogenesis in brain recovery following ischemic stroke. CD31 is mainly expressed in the endothelium and plays a key role in the generation of new blood vessels. Thus, immunofluorescence showed that the number of Smad 1/5-positive cells colocalized with ECs (CD31-positive cells) in the infarct zone was significantly increased in mice treated with Coicis Semen compared to mice treated with vehicle (*P*< 0.05, Figure 5A, 5C). Additionally, western blot analysis of cerebral protein extracts was applied to assess the levels of CD31 and TGF-β1 signaling-associated proteins. CD31, TGF-β1, ALK-1, Smad1/5 and p-Smad1/5 levels in the penumbra of the ischemic brain were elevated at 7 and 14 days after stroke, and this increase was significantly improved by Coicis Semen administration (*P* < 0.05, Figure 6). Overall, our results indicated that Coicis Semen treatment could effectively improve angiogenesis and activate the TGF-β1 pathway in MCAO mice during the recovery phase.

### Coicis semen increased cell viability after OGD/RX and upregulated angiogenesis marker protein expression in HUVECs

**Figure 7:**
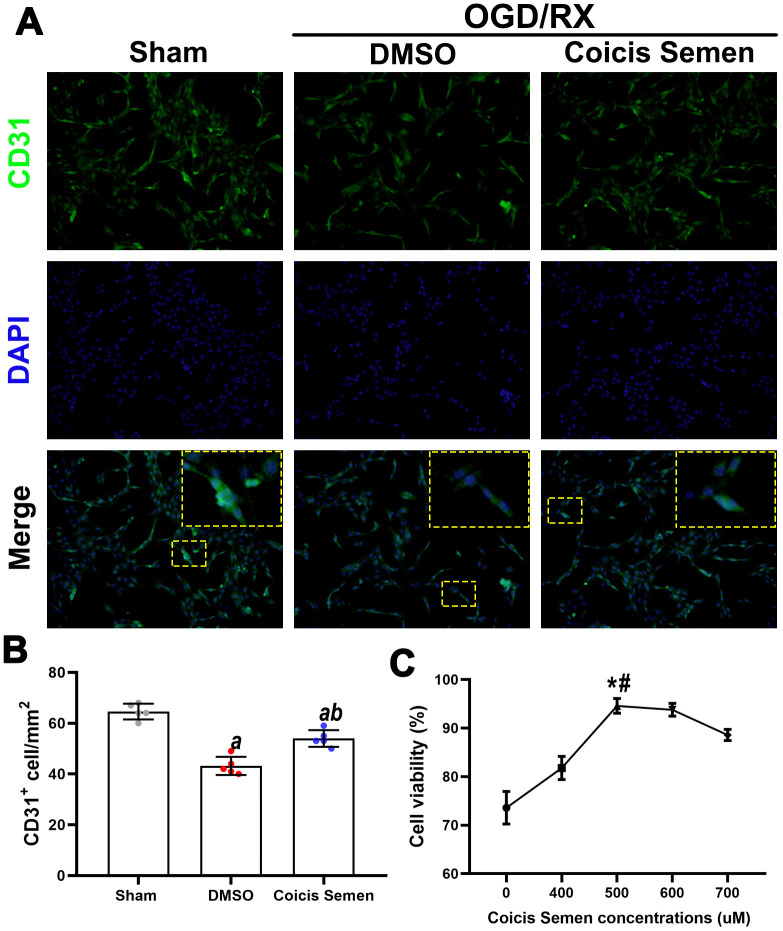
**Coicis semen increased cell viability in HUVECs after OGD/RX.** (**A**) Representative imaging showing CD31 detection in HUVECs after OGD/RX by using immunofluorescence staining. Mean ± SD. n = 5. *^a^P* < 0.05 vs. sham; ^b^*P* < 0.05 vs. DMSO. Statistical analyses were performed by one-way ANOVA followed by Tukey’s multiple comparison tests. (**B**) Quantitative analysis of CD31-positive cells. (**C**) Quantitative analysis of cell viability in cells treated with Coicis Semen at different doses. Mean ± SD. n = 5. *^a^P* < 0.05 vs. sham; ^b^*P* < 0.05 vs. DMSO; *P < 0.05 vs. Coicis Semen (400 μM); ^#^*P* < 0.05 vs. Coicis Semen (600 μM). Statistical analyses were performed by one-way ANOVA followed by Tukey’s multiple comparison tests.

ECs play a pivotal role in vascular remodeling and angiogenesis, but the role of Coicis Semen and its molecular signaling mechanism in ECs remain unknown. To address these issues, we used HUVECs to generate a stroke model by OGD/RX simulation *in vivo*. First, we performed a CCK-8 cytotoxicity assay to determine the optimal concentration of Coicis Semen to be administered to the HUVECs after OGD/RX. Coicis Semen at 400 μM, 500 μM, 600 μM and 700 μM effectively improved the relative viability of cocultured cells by comparing with DMSO group (*P* < 0.01, *P* < 0.01, *P* < 0.05, *P* < 0.05, Figure 7C), and we chose 500 μM (by compared with 400 μM, *P* < 0.01; and by compared with 600 μM, *P* < 0.05) as the optimal Coicis Semen concentration for subsequent experiments based on a previous study [24]. Then, we used immunofluorescence analysis to detect CD31 in HUVECs to confirm that Coicis Semen promoted angiogenesis in HUVECs after OGD/RX. The number of CD31-positive cells after OGD/RX was reduced in the vehicle-treated group compared to the sham group. Meanwhile, consistent with the *in vivo* results, Coicis Semen also increased the number of CD31-positive cells in the *in vitro* ischemia-reperfusion model (*P* < 0.05, Figure 7A, 7B).

### Coicis semen promoted angiogenesis via upregulation of the TGF-β1/ALK1/Smad1/5 pathway after OGD/RX *in vitro*

**Figure 8:**
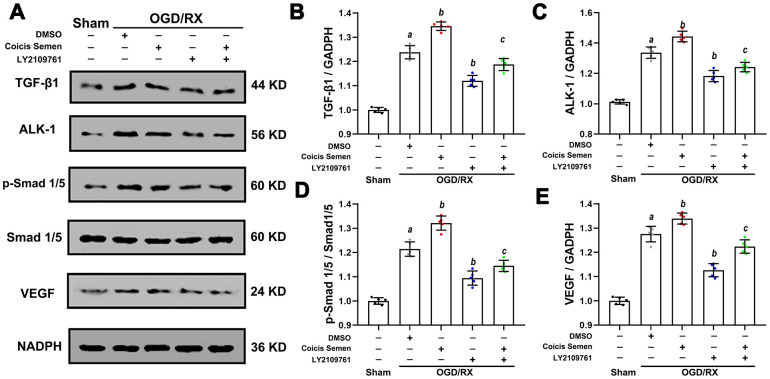
**Coicis semen promoted angiogenesis in HUVECs after OGD/RX through upregulation of the TGF-β1-ALK1-Smad1/5 signaling pathway.** (**A**) Representative western blots showing TGF-β1, ALK1, Smad1/5, p-Smad1/5 and VEGF. Quantitative analysis of TGF-β1 (**B**), ALK1 (**C**), p-Smad1/5 (**D**), and VEGF (**E**) protein expression levels in HUVECs after OGD/RX. Mean ± SD. n = 8. *^a^P* < 0.05 vs. sham; *^b^P* < 0.05 vs. DMSO + OGD/RX; *^c^P* < 0.05 vs. LY2109761 + OGD/RX. Statistical analyses were performed by one-way ANOVA followed by Tukey’s multiple comparison tests.

Based on the above results, we speculated that the neuroprotective effect of Coicis Semen is related to its antioxidative function and ability to promote angiogenesis via activation of the TGF-β1 signaling pathway. To confirm the molecular mechanism by which Coicis Semen promotes angiogenesis, we utilized LY2109761, an inhibitor of the TGF-β receptor kinase that can effectively block stimulation of p-Smad1/5 expression. Compared to that in the vehicle-treated group, the expression of TGF-β1 and its downstream target p-Smad1/5 in HUVECs after OGD/RX was downregulated after LY2109761 treatment (*P* < 0.01). However, their expression levels were upregulated by cotreatment with LY2109761 and Coicis Semen compared with those following treatment with LY2109761 alone. Then, we further measured the levels of VEGF, which is regulated by TGF-β1/ALK1/Smad1/5, and found that they were downregulated after inhibition with LY2109761 but increased by cotreatment with LY2109761 and Coicis Semen (*P* < 0.05) (Figure 8). These results strongly suggest that the protective effect of Coicis Semen on cerebral ischemia-reperfusion injury (CIRI) occurs through facilitating angiogenesis and inhibiting oxidative stress activation via upregulation of the TGF-β1/ALK1/Smad1/5 pathway.

## DISCUSSION

Stroke is a major cause of disability and death worldwide, but the agents currently used to treat stroke are not sufficient to effectively improve the recovery of stroke patients [25, 26]. Coicis Semen, a traditional Chinese medicine, exerts antioxidant and anti-inflammatory effects in many diseases, including coronary heart disease [27], systemic inflammatory disease [28], and cancer [9, 29]. In a previous study, Coicis Semen was shown to have an antioxidant effect, and oxidative stress is closely associated with the prognosis of ischemic stroke [28, 30]. This led us to speculate that Coicis Semen also protects against CIRI. Here, for the first time, we show that Coicis Semen has a neuroprotective effect against ischemic stroke by inhibiting oxidative stress activation and promoting angiogenesis. First, we successfully established CIRI models *in vivo* and *in vitro,* after which we measured the infarct volume, cerebral edema, neurological function, survival rate and behavior from 3 to 14 days following stroke *in vivo* and cell viability *in vitro*. Coicis Semen significantly increased the survival rate of MCAO mice, reduced the infarct volume, alleviated edema, improved neurological function and increased the cell viability of HUVECs after OGD/RX. Second, to explore the neuroprotective mechanisms of Coicis Semen, we detected the levels of oxidation and antioxidant indicators, ROS, SOD, GSH and MDA, in the brain and assessed BBB function as well as angiogenesis levels. Coicis Semen dramatically inhibited oxidative stress activation, alleviated BBB leakage and promoted angiogenesis in CIRI. Third, to further explore the molecular signaling mechanism by which Coicis Semen promotes angiogenesis and inhibits oxidative stress activation, we measured the expression of TGF-β1 and its downstream proteins, which have well-established effects on angiogenesis. Coicis Semen treatment significantly activated the TGF-β1/ALK1/Smad1/5 pathway in the recovery phase of stroke. Furthermore, an *in vitro* study confirmed that Coicis Semen treatment significantly increased EC viability and promoted angiogenesis via the activation of TGF-β1/ALK1/Smad1/5 signaling after ischemic injury, facilitating facility after CIRI.

In the acute phase of ischemic stroke, cerebral infarction, neurological function deficit and BBB damage are triggered by mitochondrial dysfunction, the inflammatory cascade and energy metabolism disorders, resulting in the overproduction of oxygen free radicals [31]. Accumulating evidence has confirmed that excess ROS may inhibit antioxidant enzyme activity, mediate oxidative damage to DNA/RNA, and finally induce neuronal apoptosis [32]. Although ROS at moderate concentrations are important for physiological processes, excessive ROS generation and antioxidant consumption contribute to secondary brain injury [33]. As the brain is the part of the body most susceptible to oxidative stress, several studies have found that an increase in oxygen free radical formation leads to a larger infarct volume and more severe neurologic deficit [34–36]. Moreover, antioxidant (SOD and GSH) consumption as well as malondialdehyde (MDA) accumulation directly reflect the degree of lipid peroxidation in ischemic stroke. Polysaccharides and polyphenols, major active components of Coicis Semen, are thought to have immunological, antioxidant, and anti-inflammatory effects [27, 37, 38]. Importantly, in previous studies, Coicis Semen was shown to decrease the levels of GSH and SOD and increase serum MDA levels in rats with rheumatoid arthritis [28]. Thus, in the present study, we examined the levels of ROS, SOD, GSH and MDA in the brains of MCAO mice to measure the degree of oxidative stress. We found that ischemic stroke initiated oxidative stress activation, generated excess ROS and MDA, and decreased the levels of SOD and GSH, but all of these effects were inhibited by Coicis Semen treatment. Thus, the neuroprotective effect of Coicis Semen may be related to the inhibition of oxidative stress activation during the acute phase of ischemic stroke.

Angiogenesis is a vital protective and restorative mechanism in response to ischemic stroke [39]. Occurring in the penumbra region as early as 12 h after stroke, angiogenesis was shown to last for more than 21 days following cerebral ischemia in rodent experiments [40, 41]. Angiogenesis in the ischemic boundary region after ischemic stroke contributes to the reformation of neuropil networks and restoration of blood flow and neuronal metabolism in tissue at risk [42]. Consistent with previous studies [13], our findings also confirmed that the expression of CD31 (an angiogenesis marker) gradually increased throughout a prolonged recovery period (1-14 days after MCAO). Furthermore, Coicis Semen treatment accelerated microvessel remodeling during the recovery phase of ischemic stroke (7 and 14 days after MCAO). In the recovery phase of stroke, angiogenesis is triggered by the proliferation and differentiation of vascular smooth muscle cells and the activation of matrix metalloproteinases. Additionally, the BBB, a protective barrier of the CNS, is constructed by ECs, astrocytic end-feet, pericytes, and tight junction proteins. Ischemia-induced BBB breakdown leads to cerebral edema, hemorrhage and an inflammatory cascade, resulting in secondary brain injury [43]. In this study, we found that BBB leakage gradually improved with prolonged recovery time after stroke, and Coicis Semen improved the recovery of BBB function. New blood vessel generation contributed to BBB functional recovery and improved the prognosis of stroke. Consistent with the neurological function improvement observed at 7 and 14 days in the MCAO mice, Coicis Semen not only attenuated ischemia-induced BBB leakage but also promoted angiogenesis in the penumbra of the ischemic region at 7 and 14 days after stroke.

Many molecules, including insulin-like growth factor (IGF-1), epidermal growth factor (EGF), erythropoietin (EPO) and hepatocyte growth factor (HGF), are known to regulate angiogenesis. A signaling pathway involving VEGF was identified as a key player in the regulation of angiogenesis [44]. In our study, we found that Coicis Semen could increase the levels of VEGF at 7 and 14 days after stroke and elevate the viability of ECs after OGD/RX. Many signaling pathways are involved in the regulation of VEGF. In a previous study, the TGF-β1-ALK1-Smad1/5 signaling pathway was confirmed to play an important role in angiogenesis and the regulation of VEGF in ECs and mural cells [45]. The TGF-β superfamily consists of multifunctional cytokines that exerts their effects by binding specific type I and type II serine or threonine kinase receptors and transferring intracellular Smad transcription factors to the nucleus. ALK-1 is a type І receptor of TGF-β that is mainly expressed on ECs [46]. Upon ALK-1 activation, receptor-regulated Smad1 and 5 are recruited to ALK-1 and phosphorylated by ALK-1 at two serine residues, after which they are translocated to nucleus and then stimulate EC migration, proliferation and tube formation [47]. Additionally, TGF-β1 has been reported to protect hippocampal neurons from multiple types of damage, including oxidative stress and excitotoxic and ischemia injury [48]. A recent study in models of brain ischemia demonstrated that the TGF-β1 signaling pathway is involved in brain ischemia by regulating neurogenesis and angiogenesis [49]. Our findings revealed that ischemic stroke initiated TGF-β1-ALK1-Smad1/5 pathway activation and increased the levels of VEGF, but these changes were improved by Coicis Semen treatment. To further support the effect of Coicis Semen in activating the TGF-β1-ALK1-Smad1/5 signaling pathway, LY2109761 was utilized. In *in vitro* OGD/RX experiments, we found that Coicis Semen also activated the TGF-β1-ALK1-Smad1/5 signaling pathway after its inhibition by LY2109761 in ECs. These findings suggest that Coicis Semen exerts a neuroprotective effect in the ischemic brain partly by activating the TGF-β1-ALK1-Smad1/5 pathway, subsequently improving angiogenesis and BBB function.

The central finding of our study is that Coicis Semen reduces brain infarction by inhibiting oxidative stress activation, restoring the BBB and promoting angiogenesis; these effects are related to TGF-β1-ALK1-Smad1/5 signaling pathway activation. Over recent years, most neuroprotective agents assessed in previous studies have been designed to target pathological cascades in ischemic stroke (such as anti-inflammatory glutamate antagonists), apoptosis and necrosis [50, 51]. However, most neuroprotective agents have some side effects, and clinical therapy with these agents is limited. Our current study shows that Coicis Semen, a common food in Asian countries, can protect against CIRI and may provide a rationale in support of its clinical translation.

## MATERIALS AND METHODS

### Animals

Wild-type C57BL/6J mice (n = 180, when animals that died and animals in which modeling was unsuccessful due to the absence of infarction or infarction with hemorrhage were excluded (n=77), 25-30 g) were obtained from Hubei Experimental Animal Research Center (Hubei, China; Nos. 43103740114827, 43103740014717, 43203440117027). The animals were house in an environment with controlled humidity (60 ± 5%) and temperature (21 ± 2° C) under a 12/12-h light/dark cycle and given free access to standard laboratory food and water for at least 1 week before the experiments. All animal experimental protocols were approved by the Animal Experimentation Ethics Committee of Wuhan University and conducted according to the Animal Care and Use Committee guidelines of Renmin Hospital of Wuhan University.

### Drug administration and experimental grouping

Coicis Semen (100 g; S27205, Yuanye Bio-Technology, Shanghai, China) was prepared in 100 mM dimethyl sulfoxide (DMSO) to the desired concentrations. Before MCAO, mice received Coicis Semen at different concentrations by oral administration for 28 days as previously described [27, 28]. The mice were randomly divided into the following groups: the sham, MCAO + vehicle, MCAO + Coicis Semen (50 mg/kg), MCAO + Coicis Semen (100 mg/kg), and MCAO + Coicis Semen (150 mg/kg) groups. After the appropriate doses of Coicis Semen were chosen at 3 days after MCAO, we administered Coicis Semen at these concentrations to MCAO mice for 7 and 14 days. A vehicle solution containing no Coicis Semen was administered to the MCAO + vehicle group.

### MCAO modeling and infarct volume measurement

The mice were subjected to MCAO, the method that produces injury most similar to human stroke, as previously described [12, 52]. Briefly, wild-type C57BL/6J mice were anesthetized with 5% isoflurane in O_2_ with a facemask, and the left middle cerebral artery was ligated with 6-0 monofilament nylon (Doccol, Corp., Redlands, CA, United States). After 1 h of occlusion, the monofilament nylon was removed, and reperfusion was initiated. The mice were placed on a homeothermic heating pad (TMS-202, Softron Biotechnology, Beijing China) to stabilize their body temperature at 37 ± 0.5° C. Mice in the sham group underwent the same procedure without monofilament ligation. The mice were deeply anesthetized and euthanized with an overdose of isoflurane on days 3, 7 and 14 after MCAO. The brain tissues were cut into 2-mm coronal sections and then stained in 2% 2,3,5-triphenyltetrazolium chloride (TTC) (17779, Sigma-Aldrich, United States). The infarct volume was detected and analyzed by a blinded observer using ImageJ v1.37 (NIH, Bethesda, MA, United States) as described previously [53] and normalized and is presented as a percentage of the volume in the nonischemic hemisphere to correct for edema.

### Assessment of neurological deficit

Neurological deficit scores were evaluated from 1 to 14 days after MCAO as described previously [54, 55]. The scores ranged from 0 (no observable neurological deficits) to 4 (no spontaneous motor activity and loss of consciousness) and were measured with a method from Longa EZ [56].

### Brain water content

Cerebral edema was assessed 3 days after reperfusion by the wet/dry method as previously described [57]. In brief, the brains were removed without perfusion, and the wet weights were measured on an electronic balance. Dry weights were measured after heating the specimens at 105° C for 24 h. The brain water content was calculated by using the following formula: (wet weight-dry weight)/wet weight × 100%.

### Rotarod test

The time that each mouse spent on a rotarod was measured with an accelerating rotarod (SD Instruments, San Diego, CA). Before MCAO, each mouse was trained for 9 days, and the rotation speed was increased from 4 to 8 rpm every 4 min. The time between the placement of the mouse on the pole and its falling off the rod was recorded. After the training period, the mice in each group performed the test on days 1, 3, 5, 7, and 14 after stroke. The times were calculated by averaging the times recorded for each mouse in three experiments.

### Beam-walking tests

Beam-walking tests were performed using wooden beams of cylindrical ridged beam 8 mm in diameter and the beam was 30 cm long and placed 30 cm above the bench top. The times were calculated according to the ability of the mouse to walk along the beam from one end to the other.

### Pole test

Each mouse was placed on a 50-cm vertical pole with its head upward. The time that the mouse spent on the pole before falling to the cage floor was recorded. Before MCAO, each mouse was trained for 3 days (5 trials per day). After the training period, the mice in each group were tested on days 1, 3, 5, 7, and 14 after stroke. The times were calculated by averaging the three times recorded for each mouse in three experiments.

### Measurement of ROS, SOD, GSH and MDA levels

To assess ROS production, the brain was carefully and quickly isolated, cut into 4.0-μm sections with a freezing microtome (CryoStar NX50, Thermo, USA, MA) and placed on chilled microscope slides. The samples were incubated in physiological saline solution (PSS) containing 10 μmol of dihydroethidium (DHE; Sigma-Aldrich) for 30 min at 37° C in a dark room. The brain was washed twice with PSS and placed under an automatic fluorescence microscope (BX63, Olympus Optical Ltd., Tokyo, Japan). The brain tissue was removed and immediately dissected on a cooled ice pack (−20° C) at the indicated time after reperfusion. Brain SOD, GSH and MDA levels were measured by SOD, GSH and MDA assay kits, respectively, following the manufacturer's directions (A001-3-2, A006-2-1, A005-2-1, Nanjing Jian Cheng Bioengineering Institute, Nanjing, China).

### BBB permeability assay

Evans blue (EB; 2% in PBS; Sigma-Aldrich, E2129) dye was injected via the tail vein at 4 h before the mouse was sacrificed. The entire brain tissue was homogenized and incubated in formamide (24 h, 55° C), and the absorbance of the homogenate supernatant at 620 nm was measured. The EB concentrations were normalized based on the results from brain samples from the sham-operated mice. The results were calculated using a standard curve of EB in formamide and are presented in units of micrograms per gram of brain tissue.

### VEGF ELISA

A homogenate of the infarct area of the brain tissue containing VEGF was centrifuged (1200 ×g, 10 min, 4° C), and the concentrations of VEGF in the supernatant was measured with an ELISA kit according to the manufacturer’s instructions (MMV00, R&D Systems, Minnesota, USA).

### Immunofluorescence staining

The mice were euthanized, perfused with cold PBS, and fixed with 4% paraformaldehyde for 2 days. Paraffin sections were then prepared from the brains. Before staining, the sections were deparaffinized and rehydrated, and antigen retrieval was carried out, followed by treatment with 0.3% hydrogen peroxide to quench endogenous peroxidase activity. The cells were adhered to plates before staining and washed 3 times with cold PBS. Afterward, the slices and cells were blocked with 0.1 M PBS containing 5% fetal bovine serum (FBS) and 0.3% Triton X for 1 h at room temperature. After washing, the slices were incubated at 4° C overnight with the following primary antibodies: anti-CD31 (1:200, ab28364, Abcam, Cambridge, United Kingdom) and anti-Smad 1/5 (1:200, abs130460, Absin, Beijing, China). The slices were then rinsed and incubated with Alexa594-conjugated anti-CD31 antibody (1:200, ANT030, Millipore, Billerica, MA, United States) or Alexa 488-conjugated anti-Smad1/5 antibody (1:200, ANT024, Millipore, Billerica, MA, United States) for 2 h at room temperature. After thorough rinsing, the nuclei were stained with DAPI (94010, Vector Laboratories, Burlingame, CA, United States). All slices were photographed using an automatic fluorescence microscope (BX63, Olympus Optical, Ltd., Tokyo, Japan). The numbers of immunoreactive cells in predefined areas were quantified using ImageJ software (Media Cybernetics, Inc., Rockville, MD, United States). Cells in six different fields per mouse in six mice per group were counted. All counts were conducted by blinded observers.

### *In vitro* cell culture and OGD/RX

HUVECs were obtained from the China Center for Type Culture Collection (Wuhan, China) and cultured in Dulbecco’s modified Eagle’s medium (DMEM) supplemented with 10% FBS (#13011-8611, Zhejiang Tian Hang Biotechnology, Co., Ltd., Zhejiang, China) and 1% antibiotic (GNM15140, Genome, China) in an incubator supplied with 5% CO_2_ at 37° C. Before OGD/RX injury was induced, the cultured cells in the logarithmic growth phase were rinsed twice with PBS and maintained in glucose-free DMEM. The cells were then placed into a hypoxic incubator (Binder, CB-210 hypoxia workstation) under 1% O_2_, 5% CO_2_, and 94% N_2_ for 8 h at 37° C to mimic OGD injury. The culture medium was then replaced with glucose-containing DMEM, and the cells recovered under normoxic conditions (37° C, 5% CO_2_) for 12 h (OGD restoration) as described previously [58]. Cells in the control groups without OGD were washed twice with PBS and maintained in DMEM without oxygen deprivation.

### Drug treatment and *in vitro* cell viability assay

The Cell Counting Kit (CCK)-8 assay (Dojindo Laboratories, Kumamoto, Japan) was used to assess cell viability. Briefly, HUVECs were seeded in 96-well plates containing DMEM containing 10% FBS. The TGF-β inhibitor LY2109761 (S2704, Selleckchem, Houston, TX, USA) was reconstituted in DMSO and stored at -80° C. First, HUVECs at the beginning of the OGD period were treated with LY2109761 at different concentrations (0, 0.5, 1, 1.5, and 2 μM) [45] and Coicis Semen at different concentrations (10, 400, 500, 600, and 700 μM) [24] after OGD/RX to choose the appropriate doses. Then, the medium was removed, and 10 μl of CCK-8 solution was added to each well. After 2 h of incubation at 37° C, the absorbance at 450 nm was measured using an automatic microplate reader (PerkinElmer Victor 1420, Alburgh, VT, United States).

### Western blot analysis

Total protein was extracted from HUVECs and ipsilateral brain tissue harvested after MCAO. Then, the samples were homogenized in cold RIPA buffer (C1053, Applygen, Beijing, China), and protease inhibitor cocktail (G2006, Servicebio, Wuhan, China) was added. The homogenates were centrifuged at 4° C at 10,000 × g for 30 min, and the supernatants were then harvested. The protein content was determined with a BCA kit (G2026, Servicebio, Wuhan, China). Protein samples (20 μL/lane) were separated by electrophoresis on 4-15% sodium dodecyl sulfate-polyacrylamide gels and then transferred to PVDF membranes (Millipore, Billerica, MA, United States). The membranes were then blocked in 5% nonfat milk with PBS/0.1% Tween for 1 h, followed by incubation overnight with anti-ZO-1 (1:1,000, ab96587, Abcam, Cambridge, United Kingdom), anti-Occludin (1:1,000, ab216327, Abcam, Cambridge, United Kingdom), anti-TGF-β1 (1:1,000, ab92486, Abcam, Cambridge, United Kingdom), anti-ALK-1 (1:1,000, ab108207, Abcam, Cambridge, United Kingdom), anti-Smad1/5 (1:500, ab66737, Abcam, Cambridge, United Kingdom), anti-phosphorylated (p-)Smad1/5 (1:500, #9516, Cell Signaling Technology, Boston, MA, United States), anti-VEGF (1:1,000, #2479, Cell Signaling Technology, Boston, MA, United States), and anti-CD31 (1:1,000, ab28364, Abcam, Cambridge, United Kingdom) antibodies at 4° C. After washing with PBS/0.1% Tween, the membranes were incubated with HRP-labeled secondary antibody (1:10,000, ab6721 for anti-rabbit, ab6728 for anti-mouse, Abcam, Cambridge, United Kingdom) at room temperature for 1 h. Then, ECL-A buffer and ECL-B buffer (Servicebio, Wuhan, China) were used for imaging. Images were acquired with a ChemiDoc imaging system (Bio-Rad). The relative band intensities were calculated using Quantity One v4.6.2 software (Bio-Rad Laboratories, Hercules, CA, United States) and then normalized to the control, GAPDH. All of the above experiments were performed three times.

### Statistical analysis

Data are presented as the mean ± SD. Differences were considered to be significant at P < 0.05. The normality of the distribution of all data included in these studies was analyzed using the Shapiro-Wilk test. The F-test was used to evaluate equal variances. Then, we used one-way analysis of variance (ANOVA) with Tukey's post hoc test and two-way repeated-measures ANOVA with Sidak post hoc test, followed by the least significant difference test to analyze differences using GraphPad Prism 5.0 (GraphPad Software for Science, San Diego, CA). All assessments were performed by blinded observers.

### Data availability statement

The datasets generated and/or analyzed during the current study are available from the corresponding author on reasonable request in compliance with ethical standards.

### Ethics statement

The study was approved by the Ethics Committee of Renmin Hospital of Wuhan University and performed in compliance with the ARRIVE guidelines.

## References

[1] Donnan GA, Fisher M, Macleod M, Davis SM. Stroke. Lancet. 2008; 371:1612–23. 10.1016/S0140-6736(08)60694-7 18468545

[2] Zheng Y, Wu Z, Yi F, Orange M, Yao M, Yang B, Liu J, Zhu H. By activating Akt/eNOS bilobalide B inhibits autophagy and promotes angiogenesis following focal cerebral ischemia reperfusion. Cell Physiol Biochem. 2018; 47:604–16. 10.1159/000490016 29794436

[3] Wallert M, Ziegler M, Wang X, Maluenda A, Xu X, Yap ML, Witt R, Giles C, Kluge S, Hortmann M, Zhang J, Meikle P, Lorkowski S, Peter K. α-tocopherol preserves cardiac function by reducing oxidative stress and inflammation in ischemia/reperfusion injury. Redox Biol. 2019; 26:101292. 10.1016/j.redox.2019.101292 31419755 PMC6831864

[4] Kim HW, Kim JS, Cho SI. Effect of coicis semen extract on streptozotocin-induced diabetic nephropathy rats. Kor J Herbology. 2006; 21:75–81.

[5] Yun HJ, Lee YJ, Kang MS, Baek JH. Inhibitory effect of coicis semen extract (cse) on pro-inflammatory mediatory. J Korean Oriental Pediatrics. 2009; 23:159–171.

[6] Lee YJ, Sohn YJ, Lee ES, Park JS, Kim SK. Effects of coicis semen on the hyperlipidemia in rat. Kor J Herbology. 2004; 19:129–136.

[7] Hung WC, Chang HC. Methanolic extract of adlay seed suppresses COX-2 expression of human lung cancer cells via inhibition of gene transcription. J Agric Food Chem. 2003; 51:7333–37. 10.1021/jf0340512 14640580

[8] Lu X, Liu W, Wu J, Li M, Wang J, Wu J, Luo C. A polysaccharide fraction of adlay seed (coixlachryma-jobi L.) induces apoptosis in human non-small cell lung cancer A549 cells. Biochem Biophys Res Commun. 2013; 430:846–51. 10.1016/j.bbrc.2012.11.058 23200838

[9] Song SJ, Xu RJ, Xiu LJ, Liu X, Yue XQ. Network pharmacology-based approach to investigate the mechanisms of yiyi fuzi baijiang powder in the treatment of malignant tumors. Traditional Medicine Research. 2018; 3:295–306. 10.12032/TMR201814089

[10] Ruan L, Wang B, ZhuGe Q, Jin K. Coupling of neurogenesis and angiogenesis after ischemic stroke. Brain Res. 2015; 1623:166–73. 10.1016/j.brainres.2015.02.042 25736182 PMC4552615

[11] Kawabori M, Yenari MA. Inflammatory responses in brain ischemia. Curr Med Chem. 2015; 22:1258–77. 10.2174/0929867322666150209154036 25666795 PMC5568039

[12] Zhang ZG, Zhang L, Jiang Q, Zhang R, Davies K, Powers C, van Bruggen N, Chopp M. VEGF enhances angiogenesis and promotes blood-brain barrier leakage in the ischemic brain. J Clin Invest. 2000; 106:829–38. 10.1172/JCI9369 11018070 PMC517814

[13] Hatakeyama M, Ninomiya I, Kanazawa M. Angiogenesis and neuronal remodeling after ischemic stroke. Neural Regen Res. 2020; 15:16–19. 10.4103/1673-5374.264442 31535636 PMC6862417

[14] Hayashi T, Noshita N, Sugawara T, Chan PH. Temporal profile of angiogenesis and expression of related genes in the brain after ischemia. J Cereb Blood Flow Metab. 2003; 23:166–80. 10.1097/01.WCB.0000041283.53351.CB 12571448

[15] Kanazawa M, Miura M, Toriyabe M, Koyama M, Hatakeyama M, Ishikawa M, Nakajima T, Onodera O, Takahashi T, Nishizawa M, Shimohata T. Microglia preconditioned by oxygen-glucose deprivation promote functional recovery in ischemic rats. Sci Rep. 2017; 7:42582. 10.1038/srep42582 28195185 PMC5307390

[16] Tian H, Mythreye K, Golzio C, Katsanis N, Blobe GC. Endoglin mediates fibronectin/α5β1 integrin and TGF-β pathway crosstalk in endothelial cells. EMBO J. 2012; 31:3885–900. 10.1038/emboj.2012.246 22940691 PMC3463850

[17] Hoodless PA, Wrana JL. Mechanism and function of signaling by the TGF beta superfamily. Curr Top Microbiol Immunol. 1998; 228:235–72. 10.1007/978-3-642-80481-6_10 9401209

[18] González-Núñez M, Muñoz-Félix JM, López-Novoa JM. The ALK-1/Smad1 pathway in cardiovascular physiopathology. A new target for therapy? Biochim Biophys Acta. 2013; 1832:1492–510. 10.1016/j.bbadis.2013.05.016 23707512

[19] Oh SP, Seki T, Goss KA, Imamura T, Yi Y, Donahoe PK, Li L, Miyazono K, ten Dijke P, Kim S, Li E. Activin receptor-like kinase 1 modulates transforming growth factor-beta 1 signaling in the regulation of angiogenesis. Proc Natl Acad Sci USA. 2000; 97:2626–31. 10.1073/pnas.97.6.2626 10716993 PMC15979

[20] Goumans MJ, Liu Z, ten Dijke P. TGF-beta signaling in vascular biology and dysfunction. Cell Res. 2009; 19:116–27. 10.1038/cr.2008.326 19114994

[21] Pardali E, ten Dijke P. Transforming growth factor-beta signaling and tumor angiogenesis. Front Biosci (Landmark Ed). 2009; 14:4848–61. 10.2741/3573 19482591

[22] Zhang K, Zhang Q, Deng J, Li J, Li J, Wen L, Ma J, Li C. ALK5 signaling pathway mediates neurogenesis and functional recovery after cerebral ischemia/reperfusion in rats via Gadd45b. Cell Death Dis. 2019; 10:360. 10.1038/s41419-019-1596-z 31043581 PMC6494915

[23] Jin J, Sun H, Liu D, Wang H, Liu Q, Chen H, Zhong D, Li G. LRG1 promotes apoptosis and autophagy through the TGFβ-smad1/5 signaling pathway to exacerbate ischemia/reperfusion injury. Neuroscience. 2019; 413:123–34. 10.1016/j.neuroscience.2019.06.008 31220542

[24] Hu Y, Zhou Q, Liu T, Liu Z. Coixol suppresses NF-κB, MAPK pathways and NLRP3 inflammasome activation in lipopolysaccharide-induced RAW 264.7 cells. Molecules. 2020; 25:894. 10.3390/molecules25040894 32085388 PMC7070437

[25] Malik AR, Lips J, Gorniak-Walas M, Broekaart DW, Asaro A, Kuffner MT, Hoffmann CJ, Kikhia M, Dopatka M, Boehm-Sturm P, Mueller S, Dirnagl U, Aronica E, et al. SorCS2 facilitates release of endostatin from astrocytes and controls post-stroke angiogenesis. Glia. 2020; 68:1304–16. 10.1002/glia.23778 31898841

[26] Kim Y, Lee S, Zhang H, Lee S, Kim H, Kim Y, Won MH, Kim YM, Kwon YG. CLEC14A deficiency exacerbates neuronal loss by increasing blood-brain barrier permeability and inflammation. J Neuroinflammation. 2020; 17:48. 10.1186/s12974-020-1727-6 32019570 PMC7001304

[27] Wang L, Sun J, Yi Q, Wang X, Ju X. Protective effect of polyphenols extract of adlay (Coix lachryma-jobi L. var. ma-yuen Stapf) on hypercholesterolemia-induced oxidative stress in rats. Molecules. 2012; 17:8886–97. 10.3390/molecules17088886 22836208 PMC6268808

[28] Zhang C, Zhang W, Shi R, Tang B, Xie S. Coix lachryma-jobi extract ameliorates inflammation and oxidative stress in a complete freund’s adjuvant-induced rheumatoid arthritis model. Pharm Biol. 2019; 57:792–98. 10.1080/13880209.2019.1687526 31747811 PMC6882456

[29] Son ES, Kim SH, Kim YO, Lee YE, Kyung SY, Jeong SH, Kim YJ, Park JW. Coix lacryma-jobi var. Ma-yuen stapf sprout extract induces cell cycle arrest and apoptosis in human cervical carcinoma cells. BMC Complement Altern Med. 2019; 19:312. 10.1186/s12906-019-2725-z 31729992 PMC6858790

[30] Lu Y, Li C, Chen Q, Liu P, Guo Q, Zhang Y, Chen X, Zhang Y, Zhou W, Liang D, Zhang Y, Sun T, Lu W, Jiang C. Microthrombus-targeting micelles for neurovascular remodeling and enhanced microcirculatory perfusion in acute ischemic stroke. Adv Mater. 2019; 31:e1808361. 10.1002/adma.201808361 30957932

[31] Lin TN, He YY, Wu G, Khan M, Hsu CY. Effect of brain edema on infarct volume in a focal cerebral ischemia model in rats. Stroke. 1993; 24:117–21. 10.1161/01.str.24.1.117 8418534

[32] Lorenzano S, Rost NS, Khan M, Li H, Batista LM, Chutinet A, Green RE, Thankachan TK, Thornell B, Muzikansky A, Arai K, Som AT, Pham LD, et al. Early molecular oxidative stress biomarkers of ischemic penumbra in acute stroke. Neurology. 2019; 93:e1288–98. 10.1212/WNL.0000000000008158 31455665 PMC7011868

[33] Chouchani ET, Pell VR, Gaude E, Aksentijević D, Sundier SY, Robb EL, Logan A, Nadtochiy SM, Ord EN, Smith AC, Eyassu F, Shirley R, Hu CH, et al. Ischaemic accumulation of succinate controls reperfusion injury through mitochondrial ROS. Nature. 2014; 515:431–35. 10.1038/nature13909 25383517 PMC4255242

[34] Kontos HA. Oxygen radicals in cerebral ischemia: the 2001 willis lecture. Stroke. 2001; 32:2712–16. 10.1161/hs1101.098653 11692043

[35] Zini I, Tomasi A, Grimaldi R, Vannini V, Agnati LF. Detection of free radicals during brain ischemia and reperfusion by spin trapping and microdialysis. Neurosci Lett. 1992; 138:279–82. 10.1016/0304-3940(92)90933-x 1608539

[36] Cherubini A, Polidori MC, Bregnocchi M, Pezzuto S, Cecchetti R, Ingegni T, di Iorio A, Senin U, Mecocci P. Antioxidant profile and early outcome in stroke patients. Stroke. 2000; 31:2295–300. 10.1161/01.str.31.10.2295 11022053

[37] Huang DW, Kuo YH, Lin FY, Lin YL, Chiang W. Effect of adlay (Coix lachryma-jobi L. var. ma-yuen Stapf) Testa and its phenolic components on Cu2+-treated low-density lipoprotein (LDL) oxidation and lipopolysaccharide (LPS)-induced inflammation in RAW 264.7 macrophages. J Agric Food Chem. 2009; 57:2259–66. 10.1021/jf803255p 19243096

[38] Yao Y, Zhu Y, Gao Y, Ren G. Effect of ultrasonic treatment on immunological activities of polysaccharides from adlay. Int J Biol Macromol. 2015; 80:246–52. 10.1016/j.ijbiomac.2015.06.033 26123819

[39] Krupinski J, Kaluza J, Kumar P, Kumar S, Wang JM. Role of angiogenesis in patients with cerebral ischemic stroke. Stroke. 1994; 25:1794–98. 10.1161/01.str.25.9.1794 7521076

[40] Beck H, Acker T, Wiessner C, Allegrini PR, Plate KH. Expression of angiopoietin-1, angiopoietin-2, and tie receptors after middle cerebral artery occlusion in the rat. Am J Pathol. 2000; 157:1473–83. 10.1016/S0002-9440(10)64786-4 11073808 PMC1885747

[41] Carmeliet P. Angiogenesis in life, disease and medicine. Nature. 2005; 438:932–36. 10.1038/nature04478 16355210

[42] Sun P, Zhang K, Hassan SH, Zhang X, Tang X, Pu H, Stetler RA, Chen J, Yin KJ. Endothelium-targeted deletion of microRNA-15a/16-1 promotes poststroke angiogenesis and improves long-term neurological recovery. Circ Res. 2020; 126:1040–57. 10.1161/CIRCRESAHA.119.315886 32131693 PMC7172020

[43] Venkat P, Chopp M, Chen J. Blood-brain barrier disruption, vascular impairment, and ischemia/reperfusion damage in diabetic stroke. J Am Heart Assoc. 2017; 6:e005819. 10.1161/JAHA.117.005819 28572280 PMC5669184

[44] Carmeliet P. Mechanisms of angiogenesis and arteriogenesis. Nat Med. 2000; 6:389–95. 10.1038/74651 10742145

[45] Oh MK, Kim IS. Involvement of placental growth factor upregulated via TGF-β1-ALK1-Smad1/5 signaling in prohaptoglobin-induced angiogenesis. PLoS One. 2019; 14:e0216289. 10.1371/journal.pone.0216289 31034502 PMC6488081

[46] Roelen BA, van Rooijen MA, Mummery CL. Expression of ALK-1, a type 1 serine/threonine kinase receptor, coincides with sites of vasculogenesis and angiogenesis in early mouse development. Dev Dyn. 1997; 209:418–30. 10.1002/(SICI)1097-0177(199708)209:4<418::AID-AJA9>3.0.CO;2-L 9264265

[47] Goumans MJ, Valdimarsdottir G, Itoh S, Lebrin F, Larsson J, Mummery C, Karlsson S, ten Dijke P. Activin receptor-like kinase (ALK)1 is an antagonistic mediator of lateral TGFbeta/ALK5 signaling. Mol Cell. 2003; 12:817–28. 10.1016/s1097-2765(03)00386-1 14580334

[48] Cho H, Joo Y, Kim S, Woo RS, Lee SH, Kim HS. Plasminogen activator inhibitor-1 promotes synaptogenesis and protects against aβ(1-42)-induced neurotoxicity in primary cultured hippocampal neurons. Int J Neurosci. 2013; 123:42–9. 10.3109/00207454.2012.724127 22937735

[49] Ferrer I, Friguls B, Dalfó E, Planas AM. Early modifications in the expression of mitogen-activated protein kinase (MAPK/ERK), stress-activated kinases SAPK/JNK and p38, and their phosphorylated substrates following focal cerebral ischemia. Acta Neuropathol. 2003; 105:425–37. 10.1007/s00401-002-0661-2 12677442

[50] Liu NN, Deng WL, Wu CJ, Feng YY, Ma XW, Li Q. Effect of jianpi jiedu recipe on angiogenesis and the pten/pi3k/akt signaling pathway in the course of helicobacter pylori-induced gastric cancer in c57bl/6 mice. Traditional Medicine Research. 2018; 3:29–39.

[51] Li FZ, Zhang YN, Zhang T, Liu L, Wang P. Efficacy and safety of traditional chinese medicine kidney-nourishing formula for alzheimer's disease in comparison with donepezil: A systematic review and meta-analysis. Traditional Medicine Research. 2019; 3:838–848.

[52] Zhang RL, Chopp M, Zhang ZG, Jiang Q, Ewing JR. A rat model of focal embolic cerebral ischemia. Brain Res. 1997; 766:83–92. 10.1016/s0006-8993(97)00580-5 9359590

[53] Wang H, Zheng X, Jin J, Zheng L, Guan T, Huo Y, Xie S, Wu Y, Chen W. LncRNA MALAT1 silencing protects against cerebral ischemia-reperfusion injury through miR-145 to regulate AQP4. J Biomed Sci. 2020; 27:40. 10.1186/s12929-020-00635-0 32138732 PMC7059719

[54] Gu L, Xiong X, Zhang H, Xu B, Steinberg GK, Zhao H. Distinctive effects of T cell subsets in neuronal injury induced by cocultured splenocytes in vitro and by in vivo stroke in mice. Stroke. 2012; 43:1941–46. 10.1161/STROKEAHA.112.656611 22678086 PMC3506376

[55] Han RQ, Ouyang YB, Xu L, Agrawal R, Patterson AJ, Giffard RG. Postischemic brain injury is attenuated in mice lacking the beta2-adrenergic receptor. Anesth Analg. 2009; 108:280–87. 10.1213/ane.0b013e318187ba6b 19095863 PMC3661414

[56] Longa EZ, Weinstein PR, Carlson S, Cummins R. Reversible middle cerebral artery occlusion without craniectomy in rats. Stroke. 1989; 20:84–91. 10.1161/01.str.20.1.84 2643202

[57] Hatashita S, Hoff JT, Salamat SM. Ischemic brain edema and the osmotic gradient between blood and brain. J Cereb Blood Flow Metab. 1988; 8:552–59. 10.1038/jcbfm.1988.96 3392116

[58] Tian F, Zhou AX, Smits AM, Larsson E, Goumans MJ, Heldin CH, Borén J, Akyürek LM. Endothelial cells are activated during hypoxia via endoglin/ALK-1/SMAD1/5 signaling in vivo and in vitro. Biochem Biophys Res Commun. 2010; 392:283–88. 10.1016/j.bbrc.2009.12.170 20060813

